# Single-Cell Expression Landscape of SARS-CoV-2 Receptor *ACE2* and Host Proteases in Normal and Malignant Lung Tissues from Pulmonary Adenocarcinoma Patients

**DOI:** 10.3390/cancers13061250

**Published:** 2021-03-12

**Authors:** Guangchun Han, Ansam Sinjab, Kieko Hara, Warapen Treekitkarnmongkol, Patrick Brennan, Kyle Chang, Elena Bogatenkova, Beatriz Sanchez-Espiridion, Carmen Behrens, Luisa M. Solis, Boning Gao, Luc Girard, Jianjun Zhang, Boris Sepesi, Tina Cascone, Lauren A. Byers, Don L. Gibbons, Jichao Chen, Seyed Javad Moghaddam, Edwin J. Ostrin, Paul Scheet, Junya Fujimoto, Jerry Shay, John V. Heymach, John D. Minna, Steven Dubinett, Ignacio I. Wistuba, Christopher S. Stevenson, Avrum E. Spira, Linghua Wang, Humam Kadara

**Affiliations:** 1 Department of Genomic Medicine, The University of Texas MD Anderson Cancer Center, Houston, TX 77030, USA; ghan1@mdanderson.org; 2 Department of Translational Molecular Pathology, The University of Texas MD Anderson Cancer Center, Houston, TX 77030, USA; asinjab@mdanderson.org (A.S.); kkobayashi2@mdanderson.org (K.H.); wtreekit@mdanderson.org (W.T.); bsanchez2@mdanderson.org (B.S.-E.); lmsolis@mdanderson.org (L.M.S.); jfujimot@mdanderson.org (J.F.); iiwistuba@mdanderson.org (I.I.W.); 3 Pathology Department, The University of Texas MD Anderson Cancer Center, Houston, TX 77030, USA; pbrennan@mdanderson.org (P.B.); ebogatenkova@mdanderson.org (E.B.); 4 Guardant Health, Redwood City, CA 94063, USA; kchang@guardanthealth.com; 5 Department of Thoracic, Head and Neck Medical Oncology, The University of Texas MD Anderson Cancer Center, Houston, TX 77030, USA; cbehrens@mdanderson.org (C.B.); jzhang20@mdanderson.org (J.Z.); tcascone@mdanderson.org (T.C.); lbyers@mdanderson.org (L.A.B.); dlgibbon@mdanderson.org (D.L.G.); jheymach@mdanderson.org (J.V.H.); 6 Hamon Center for Therapeutic Oncology Research, University of Texas Southwestern, Dallas, TX 75390, USA; boning.gao@utsouthwestern.edu (B.G.); luc.girard@UTSouthwestern.edu (L.G.); john.minna@utsouthwestern.edu (J.D.M.); 7 Department of Cardiovascular and Thoracic Surgery, The University of Texas MD Anderson Cancer Center, Houston, TX 77005, USA; bsepesi@mdanderson.org; 8 Department of Pulmonary Medicine, The University of Texas MD Anderson Cancer Center, Houston, TX 77030, USA; jchen16@mdanderson.org (J.C.); smoghadd@mdanderson.org (S.J.M.); 9 Department of General Internal Medicine, The University of Texas MD Anderson Cancer Center, Houston, TX 77030, USA; ejostrin@mdanderson.org; 10 Department of Epidemiology, The University of Texas MD Anderson Cancer Center, Houston, TX 77230, USA; pascheet@mdanderson.org; 11 Department of Cell Biology, University of Texas Southwestern, Dallas, TX 75390, USA; jerry.shay@utsouthwestern.edu; 12 Department of Medicine, The University of California Los Angeles, Los Angeles, CA 90095, USA; sdubinett@mednet.ucla.edu; 13 Lung Cancer Initiative at Johnson and Johnson, Cambridge, MA 02142, USA; csteve22@its.jnj.com (C.S.S.); aspira@its.jnj.com (A.E.S.); 14 Section of Computational Biomedicine, Boston University, Boston, MA 02215, USA

**Keywords:** COVID-19, lung neoplasms, alveolar epithelial cells, single-cell RNA sequencing

## Abstract

**Simple Summary:**

The coronavirus disease 2019 (COVID-19) pandemic continues to spread rapidly on a global scale. When presenting with severe respiratory complications, COVID-19 results in markedly high death rates, particularly among patients with comorbidities such as cancer. Motivated by the ongoing global health crisis, we leveraged a growing in-house cohort of pulmonary tissues from lung cancer patients to analyze, at high resolution, the expression of host proteins implicated in the entryway of severe acute respiratory syndrome coronavirus 2 (SARS-CoV-2) into lung epithelial cells. Our results identify key pathways in lung pathobiology and inflammation that offer the potential to identify novel markers and therapeutic targets that can be repurposed for clinical management of COVID-19, particularly among lung cancer patients, a population that represents over half a million individuals in the United States alone.

**Abstract:**

The novel coronavirus SARS-CoV-2 is the causative agent of the COVID-19 pandemic. Severely symptomatic COVID-19 is associated with lung inflammation, pneumonia, and respiratory failure, thereby raising concerns of elevated risk of COVID-19-associated mortality among lung cancer patients. Angiotensin-converting enzyme 2 (ACE2) is the major receptor for SARS-CoV-2 entry into lung cells. The single-cell expression landscape of *ACE2* and other SARS-CoV-2-related genes in pulmonary tissues of lung cancer patients remains unknown. We sought to delineate single-cell expression profiles of *ACE2* and other SARS-CoV-2-related genes in pulmonary tissues of lung adenocarcinoma (LUAD) patients. We examined the expression levels and cellular distribution of *ACE2* and SARS-CoV-2-priming proteases *TMPRSS2* and *TMPRSS4* in 5 LUADs and 14 matched normal tissues by single-cell RNA-sequencing (scRNA-seq) analysis. scRNA-seq of 186,916 cells revealed epithelial-specific expression of *ACE2*, *TMPRSS2*, and *TMPRSS4*. Analysis of 70,030 LUAD- and normal-derived epithelial cells showed that *ACE2* levels were highest in normal alveolar type 2 (AT2) cells and that *TMPRSS2* was expressed in 65% of normal AT2 cells. Conversely, the expression of *TMPRSS4* was highest and most frequently detected (75%) in lung cells with malignant features. *ACE2*-positive cells co-expressed genes implicated in lung pathobiology, including COPD-associated *HHIP*, and the scavengers *CD36* and *DMBT1*. Notably, the viral scavenger *DMBT1* was significantly positively correlated with *ACE2* expression in AT2 cells. We describe normal and tumor lung epithelial populations that express SARS-CoV-2 receptor and proteases, as well as major host defense genes, thus comprising potential treatment targets for COVID-19 particularly among lung cancer patients.

## 1. Introduction

In late December 2019, an outbreak of lung pneumonia initially of unknown cause was reported in China [1]. This emerging disease, termed coronavirus disease 2019 (COVID-19), was soon thereafter attributed to infection with the novel zoonotically-transmitted coronavirus severe acute respiratory syndrome coronavirus 2 (SARS-CoV-2) [2]. On 11 March 2020, COVID-19 was declared a rapidly spreading global pandemic by the World Health Organization. By March 2021, SARS-CoV-2 had infected more than 118 million people worldwide, including 28 million cases and 520,000 deaths in the United States.

The clinical presentation of COVID-19 is diverse, ranging from asymptomatic infection and mild upper respiratory illness to pneumonia, acute respiratory distress syndrome (ARDS), respiratory failure, and death [1]. Recent clinical reports have suggested that old age and comorbidities such as cardiovascular disease, COPD, and cancer are risk factors for COVID-19-associated severe pneumonia and death [1]. Notably, lung cancer was found to correlate with adverse outcomes in patients with COVID-19 [3]. This has raised key questions in the clinical management of patients with both COVID-19 and lung malignancy and warrants a deeper knowledge of the yet unknown pathological mechanisms and effects of SARS-CoV-2 infection in patients with lung cancer. 

Recent studies have shown that SARS-CoV-2 infects airway cells by binding its spike protein to angiotensin-converting enzyme 2 encoded by gene *ACE2*, the same receptor used by SARS-CoV [4,5,6,7]. *ACE2* had been shown to mediate important roles in lung function, including protection from lung injury caused by SARS-CoV infection [8] and inhibition of angiogenesis in lung cancer [9]. Despite these insights, and the supposable heightened risk of lung cancer patients for COVID-19-associated mortality, the expression of SARS-CoV-2-related genes in lung tumor and uninvolved tissue is still poorly understood. 

To fill these voids, we leveraged our ongoing efforts in a single-cell transcriptomic analysis of 186,916 cells, including a large number of epithelial cells (*n* = 70,030) derived from 5 lung adenocarcinomas (LUADs) and 14 matching uninvolved normal lung tissues. We show epithelial-specific expression patterns for *ACE2* as well as *TMPRSS2* and *TMPRSS4*, serine proteases with emerging roles in SARS-CoV-2 priming [7,10]. Among all lung epithelial subsets, we find the highest expression of *ACE2* in alveolar type 2 (AT2) and malignant-enriched subsets and of *TMPRSS2* and *TMPRSS4* in AT2 and malignant-enriched cell populations, respectively. *ACE2*-positive AT2 cells also expressed genes with important yet understudied roles in lung pathobiology. Our study provides a comprehensive overview of the expression of *ACE2*, SARS-CoV-2-priming proteases, as well as host defense and scavenging genes in the malignant lung and nearby epithelium that may constitute targets that can be repurposed for the clinical management of COVID-19 in LUAD patients. 

Some of the results of this study have been previously reported in the form of an online preprint [11].

## 2. Methods

Additional descriptions of methods can be found in the Online Data Supplement.

### 2.1. Lung Tissue Processing and Single-Cell RNA-Sequencing (scRNA-Seq)

Five patients undergoing surgical resection for primary early-stage LUAD (I-IIIA) were carefully selected for the derivation of two to three uninvolved normal lung samples for single-cell analysis (*n* = 19 samples, Table S1). Samples were obtained from banked or residual specimens from patients evaluated at the University of Texas MD Anderson Cancer Center. Following tissue digestion and red blood cell removal, cells were sorted (by fluorescent-activated cell sorting) into viable singlets and, in samples from Patients 2 to 5, into separate viable epithelial (EPCAM+) and nonepithelial (EPCAM−) fractions. Single-cell gene expression libraries were generated from 35 sorted fractions using the 10× Genomics platform (Pleasanton, CA, USA) and sequenced on the Illumina NovaSeq 6000 platform (San Diego, CA, USA; Online Data Supplement).

### 2.2. scRNA-Seq Data Analysis

Raw scRNA-seq data were preprocessed, demultiplexed, and aligned to human GRCh38 reference and feature-barcodes generated using CellRanger (10× Genomics, Pleasanton, CA, USA; version 3.0.2). Details of quality control, including quality check, data filtering, identification and removal of cellular debris, doublets and multiplets, and batch effect evaluation and correction, are found in the Online Data Supplement. Following quality filtering, a total of 186,916 cells were retained for downstream analysis. Raw unique molecular identifier (UMI) counts were log normalized and used for principal component analysis. We applied Seurat [12] for unsupervised clustering analysis and Uniform Manifold Approximation and Projection (UMAP) [13] for dimensionality reduction and visualization. Lung and airway subcluster lineage (e.g., of *EPCAM*+ and AT2) was defined based on the enrichment of canonical marker genes as well as top-ranked differentially expressed genes (DEGs) in each cluster using the FindAllMarkers function in the Seurat R package. We also applied the single-cell consensus clustering (SC3) approach [14] for unsupervised clustering analysis, with default parameters independent of cell lineage annotation. 

### 2.3. Statistical Analysis

All statistical analyses were performed using R package version 3.6.0. DEG analysis (e.g., between *ACE2*-positive and *ACE2*-negative cells) was calculated using the FindMarkers function in R. Pseudobulk gene expression values for defined cell clusters were calculated by computing the mean expression of each gene across all cells in a specific cluster. Pearson’s correlation analysis was used to identify genes significantly correlated with *ACE2* expression or with an AT2 meta-score. All statistical significance testing was two-sided, and results were considered statistically significant at *p-*values < 0.05. The Benjamini–Hochberg method was applied to control the false discovery rate (FDR) in multiple comparisons (e.g., DEG analysis) and to calculate adjusted *p-*values (*q*-values).

## 3. Results

### 3.1. Single-Cell Decoding of ACE2 Expression and SARS-CoV-2 Priming Proteases in Lung Tissues of LUAD Patients

**Figure 1:**
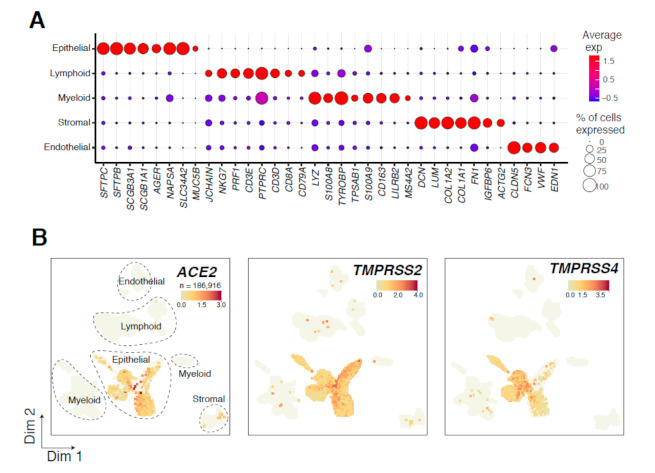
Overview of 186,916 tumor- and normal-derived lung cells analyzed by scRNA-seq. (**A**) Bubble plot show-ing the expression of markers indicative of major lineages in the lung ecosystem. Both the fraction of cells expressing (indicated by the size of the circle) as well as their scaled expression levels (indicated by the color of the circle) are shown. (**B**) Uniform manifold approximation and projection (UMAP) embedding of cells from tumor and multiple nor-mal samples per patient (19 samples from 5 LUAD patients). Cells are colored by expression level of *ACE2*, *TMPRSS2*, or *TMPRSS4*.

We performed a single-cell analysis of normal lung tissues and matched early-stage LUADs from five patients using droplet-based scRNA-seq. In the first patient, we attained a limited fraction of epithelial (*EPCAM*+) cells by an unbiased analysis of two normal lung tissues and one LUAD (~4%, *n* = 624 cells), in line with studies of other organs [15]. To better capture lung epithelial cells, we performed sequencing of cells with enriched (by sorting for EPCAM) epithelial subsets from three normal lung tissues and a matched LUAD each from four additional patients. In total, 186,916 cells, 70,030 of which were epithelial, from the 5 LUADs and 14 uninvolved normal lung tissues were retained for analysis following quality control. Based on canonical gene expression markers, cells clustered into distinct epithelial, endothelial, myeloid, lymphoid, or stromal cell clusters (Figure 1A). Prompted by the ongoing COVID-19 pandemic caused by infection with the novel coronavirus SARS-CoV-2, we leveraged our unique LUAD and normal lung tissue scRNA-seq dataset to interrogate at single-cell resolution lung expression patterns of the SARS-CoV-2 receptor *ACE2*, as well as *TMPRSS2* and *TMPRSS4*, two related membrane-bound serine proteases recently shown to be crucial for SARS-CoV-2 spike protein priming upon entry [7,10]. We found that all three SARS-CoV-2-related genes were nearly restricted to the epithelial compartment (Figure 1B), including when considering only cycling cells (Figure S1). These findings suggest that SARS-CoV-2 receptor and priming proteases are restricted to epithelial cells in the ecosystem of the normal and malignant lung.

### 3.2. Expression Patterns of ACE2, TMPRSS2, and TMPRSS4 in Normal and Malignant-Enriched Epithelial Subsets

**Figure 2:**
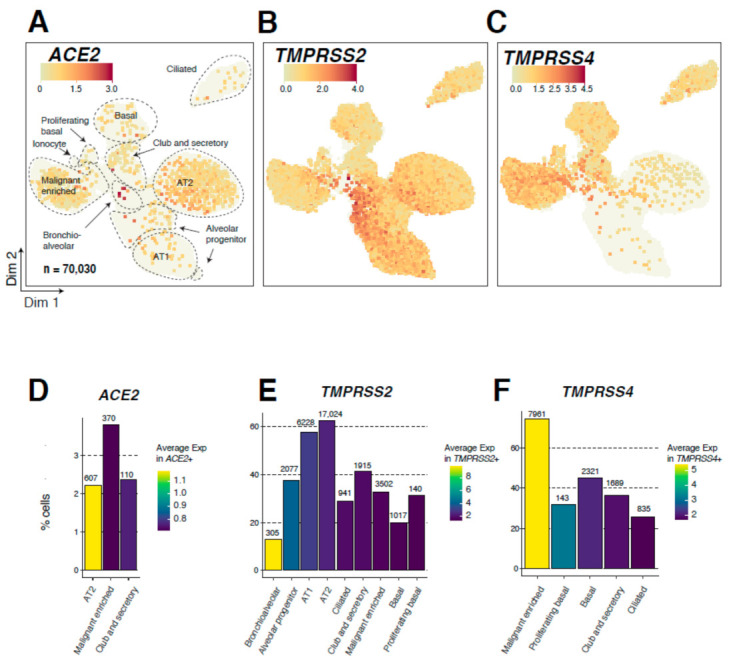
Single-cell expression analysis of *ACE2, TMPRSS2*, and *TMPRSS4* in 70,030 lung epithelial cells. (**A**–**C**) Uniform Manifold Approximation and Projection (UMAP) plots showing epithelial subclusters and expression of ACE2 (**A**), *TMPRSS2* (**B**), and *TMPRSS4* (**C**) in epithelial subclusters. Cells are colored by the expression level of each gene. AT1: alveolar type 1 cells; AT2: alveolar type 2 cells. (**D**–**F**) Bar plots showing the fraction of cells (percentage of each subcluster) expressing *ACE2* (**D**), *TMPRSS2* (**E**), and *TMPRSS4* (**F**) among airway lineage clusters with the highest fractions of cells positive for each gene (2% cutoff applied). The absolute number of cells positive for each gene and within each analyzed subcluster are indicated on top of the corresponding bars. Color indicates average expression in cells positive for the gene of interest.

We then probed expression patterns of the three genes among the 70,030 lung epithelial cells (5 tumor samples, *n* = 13,098; 14 normal lung tissues, *n* = 56,932) (Figure 2A, Table S2). We found on average 9307 UMIs and 2616 genes per epithelial cell (Table S2). Clustering analysis identified multiple airway lineages, including alveolar type 1 (AT1; *n* = 10,775), AT2 (*n* = 27,235), club and secretory (*n* = 4624), ciliated (*n* = 3247), as well as basal (*n* = 5119), including proliferative (*n* = 447) cells (Figure 2A and Figure S2A, Table S2). We also identified rare or transitory subpopulations such as bronchioalveolar cells, alveolar progenitors [16,17], and the *CFTR*-expressing pulmonary ionocytes, which represent a novel lung epithelial cell type that was recently identified in mouse and human airways [18,19,20] (Figure 2A). Additionally, a malignant-enriched cluster that was mostly derived from LUAD specimens clustered distinctly from cells of uninvolved lung tissues and exhibited high expression of tumor markers (e.g., *CEACAM5*), as well as comprised mixed-lineage genes in line with previous studies [21] (Figure S2A,B). We found that the fraction of *ACE2*-expressing cells among all lung epithelial cells was low (*n* = 1242, 1.8%, Table S3), albeit being the highest in AT2, club/secretory, and malignant subsets (Figure 2A). In comparison, *TMPRSS2* displayed a more ubiquitous epithelial expression pattern, whereby its fraction was the highest in alveolar subsets (Figure 2B), and *TMPRSS4* was largely abundant in cells of the malignant-enriched cluster (Figure 2C). Further quantification revealed that the highest fractions of *ACE2*-expressing cells were found in the malignant-enriched (3.5%), AT2 (2.2%), and club/secretory (2.4%) cell clusters (Figure 2D). Among those clusters with an absolute number of cells >50 and >2% *ACE2*-positive cells, AT2 cells exhibited the highest expression of the SARS-CoV-2 receptor (Figure 2D). The frequency of *TMPRSS2*-expressing epithelial cells was the highest among alveolar subclusters, including AT2 (62.5%), AT1 (57.8%), and alveolar progenitor (37.7%) cells, while cells of the malignant-enriched cluster displayed lower levels of *TMPRSS2* (Figure 2E). Interestingly, the other SARS-CoV-2-priming protease, *TMPRSS4*, was distinctively expressed in cells of the malignant-enriched cluster and with the greatest abundance (74.6%) and highest expression levels compared to other epithelial subclusters (Figure 2F)**.** We further interrogated tumor-associated expression patterns of these SARS-2-CoV-related host genes using bulk expression data of 52 LUAD and normal lung pairs from the TCGA cohort [22]. In line with our scRNA-seq data, *TMPRSS2* was significantly downregulated in LUADs versus matched normal lung tissues, and, in sharp contrast, *TMPRSS4* was largely upregulated in the LUADs (both *p* < 10^−7^; Figure S2C). Next, we examined *ACE2* expression in subclusters of AT2 cells (Figure S3A). We found that the same AT2 subclusters harbored the highest average expression (AT2_c2) as well as the highest fraction of cells (AT2_c3) positive for all *ACE2*, *TMPRSS2*, and *TMPRSS4* (Figure S3B–D), suggesting co-expression patterns for these SARS-CoV-2 genes in specific AT2 subsets. We also analyzed *ACE2* expression in AT2 and malignant-enriched clusters based on whether patients in our single-cell cohort were on antihypertensive treatments (Table S1), since earlier studies have shown that drugs such as losartan may impact *ACE2* expression levels [23]. Interestingly, while losartan-treated patients had lower fractions of *ACE2*-expressing AT2 cells or cells of the malignant-enriched cluster, expression levels in these subsets were significantly higher compared to their counterparts from the untreated patients (*p* < 3 × 10^−35^; Figure S4A,B). Taken together, these data describe tumor- and lung-pertinent expression patterns of the major SARS-CoV-2 receptor *ACE2* and priming proteases *TMPRSS2* and *TMPRSS4* in LUAD patients.

### 3.3. Genes Co-Expressed with ACE2 in Lung Epithelial Cells

**Figure 3:**
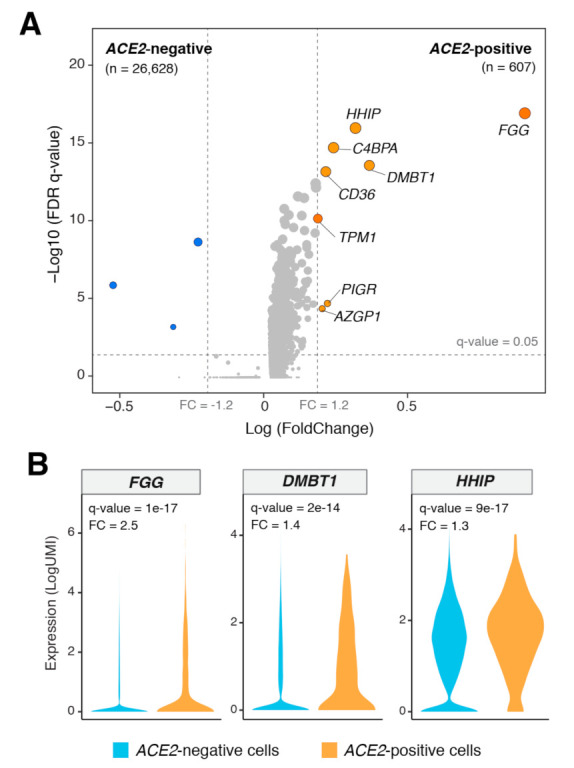
Differentially expressed genes between *ACE*2-positive and -negative AT2 cells. (**A**) Volcano plot showing significantly differentially expressed genes (DEGs) between ACE2-positive (*n* = 607) and -negative (*n* = 26,628) AT2 cells. A cutoff of absolute gene expression (fold-change: >1.2) and a FDR (*q*-value < 0.05) were applied to identify the DEGs. Blue indicates downregulation, and orange indicates upregulation. (**B**) Violin plots showing the significant upregulation of *FGG, DMBT1,* and *HHIP* genes in *ACE2*-positive compared with -negative AT2 cells. FC: Fold change.

We next sought to identify differentially expressed genes (DEGs) in AT2 cells of LUAD patients based on *ACE2* expression. Cutoffs of absolute gene expression (fold-change: >1.2) and a FDR (*q*-value < 0.05) were applied to select DEGs between *ACE2*-expressing (*n* = 607) and *ACE2*-absent (*n* = 26,628) AT2 cells. Among genes upregulated in *ACE2*-expressing AT2 cells, we identified genes that are highly pertinent to lung epithelial biology and disease such as *HHIP* (lung branching and COPD [24,25]), *FGG* (fibrosis, pneumonia, and inflammation [26,27]), and *C4BPA* (complement system, pneumonia, and infection [28,29]) (Figure 3A,B). In addition, we found that *ACE2*-expressing AT2 cells exhibited significantly a higher expression of scavengers such as *CD36* [30], as well as *DMBT1*, a pattern recognition receptor that plays crucial roles in host defense against bacterial and viral pathogens, including influenza A and human immune deficiency virus 1 (HIV-1) [31] (Figure 3A,B). Interestingly, *DMBT1* abundance and expression were markedly and distinctly highest in AT2 cells relative to other lung cell subsets (Figure S5A). *DMBT1* expression in AT2 cells was also higher compared to each of *BSG* (also known as *CD147*), which was recently suggested to represent a potential alternate route of entry for SARS-CoV-2 [32], and other coronavirus receptors such as the MERS-CoV receptor *DPP4* [33] (Figure S5A). Notably, both *DMBT1* and *BSG* (albeit to a lesser extent), but not *DPP4*, exhibited a single-cell expression pattern that resembled that of *ACE2* across the different AT2 subclusters (Figure S5B).

**Figure 4:**
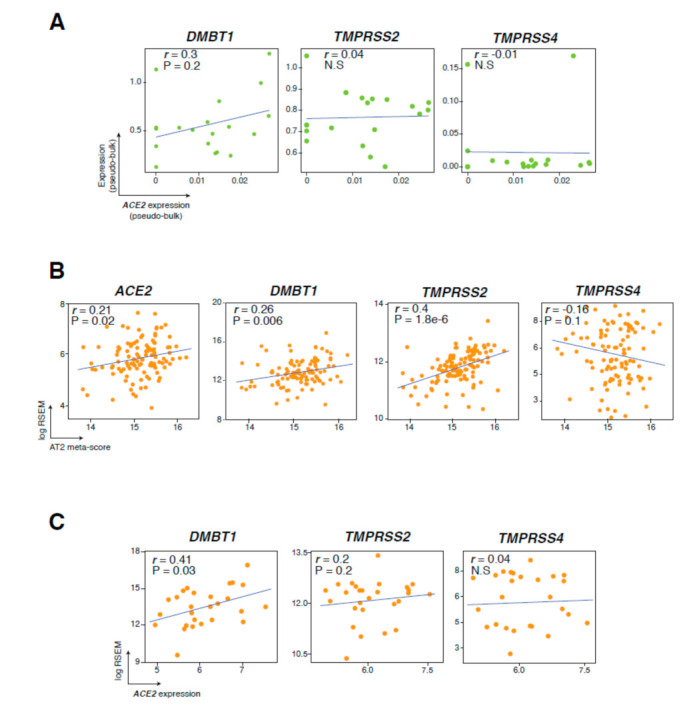
Expression of the viral scavenger *DMBT1* correlates with that of *ACE2* in lung AT2 cells. (**A**) Correlation between *ACE2* and *DMBT1, TMPRSS2,* and *TMPRSS4* expression in pseudobulk data from this study. (**B**) Scatter plots showing significant correlations between estimated AT2 cell fractions and *ACE2*, *DMBT1, TMPRSS2,* and *TMPRSS4* in TCGA normal lung samples. (**C**) Significant correlation between *ACE2* and *DMBT1* in TCGA normal lung samples with high AT2 cell fractions (meta-score > 15.48). Correlations were statistically analyzed using Pearson’s correlation coefficient.

Our findings on the significant associations between *ACE2* and *DMBT1* prompted us to probe the correlation of both genes in the AT2 compartment. We performed a pseudobulk analysis of the AT2 cluster (by sample) in our cohort and found that *DMBT1* and *ACE2* exhibited a trend of correlation (R = 0.3), albeit not reaching statistical significance (Figure 4A). We also noted nonsignificant positive and negative correlations between *ACE2* on the one hand and *TMPRSSS2* and *TMPRSS4* on the other, respectively (Figure 4A). To further interrogate those findings in larger cohorts, we performed a deconvolution analysis of bulk RNA-seq data from the TCGA LUAD cohort to estimate the abundance of AT2 cells in normal lung tissues (*n* = 110). *ACE2, DMBT1,* and *TMPRSS2* expression levels significantly and positively correlated with AT2 fractions (indicated by the AT2 meta-score, *p* < 0.05) (Figure 4B). In contrast, *TMPRSS4* expression was negatively correlated with AT2 fractions (Figure 4B), consistent with our observations of its abundance in tumors. We observed the same trends when separately analyzing stage I LUADs or stage II and III LUADs combined (Figure S6). Interestingly, the correlation between AT2 cell fractions and expression of *ACE2* and *DMBT1* was statistically significant only in stage I LUADs only (*p* < 0.05 and *p* = 0.01, respectively) (Figure S6). Further, among TCGA normal lung tissues with high AT2 fractions (meta-score > 15.48; *n* = 27), *DMBT1* expression significantly and positively correlated with *ACE2* (R = 0.41, *p* < 0.05) (Figure 4C). We did not find any significant correlation between *ACE2* and *TMPRSS2* or *TMPRSS4* in the TCGA lung tissues by deconvolution analysis (Figure 4C). Our findings point to specific *ACE2*-expressing AT2 cells in the lungs of LUAD patients that also co-express other pathogen receptors and scavengers (e.g., *DMBT1*), thereby possibly representing a minute subpopulation with unique host defense properties and functions.

## 4. Discussion

We interrogated, by single-cell RNA-sequencing, 186,916 lung cells, including a large number of epithelial cells (*n* = 70,030), from tumor and multiple matched normal tissues to comprehensively examine abundance and expression patterns of the SARS-CoV-2 receptor *ACE2* and the pathogen’s priming proteases in the lungs of LUAD patients. While *ACE2* was expressed in a low fraction of lung epithelial cells (roughly 1.8%), its levels among all lung cell subsets were highest in cells of AT2 and in malignant-enriched clusters. We also found, in alveolar and malignant-enriched subsets, the highest fractions of cells expressing SARS-CoV-2-priming proteases *TMPRSS2* and *TMPRSS4*, respectively, as well as increased expression levels of those genes. *ACE2*-positive AT2 cells co-expressed genes with crucial roles in lung pathological conditions such as COPD, pneumonia, and pathogen infection, including the viral binding scavenger *DMBT1*. We also found that *DMBT1* positively correlated with AT2 cell fractions and with *ACE2* itself within the AT2 compartment. Our findings suggest that cells expressing SARS-CoV-2 receptors and coreceptors in malignant lungs and surrounding normal tissues are relatively scarce, and they exhibit unique molecular and biological features that are pertinent to antiviral and host defense functions by the lung epithelium.

Our findings on the expression of *ACE2* in AT2 cells are in line with previous studies [34,35]. Hamming et al. showed the abundant immunohistochemical expression of ACE2 protein in AT2 cells in the lung [35]. *Ace2* was shown to not only be expressed in AT2 cells in the mouse lung but also to regulate alveolar epithelial cell survival [36]. Of note, Mossel and colleagues demonstrated that SARS-CoV, by binding to ACE2, replicates primarily in AT2 and not AT1 cells [34]. AT2 cells were also shown to be primarily responsible for an innate immune response in the lungs against SARS-CoV infection [37]. Interestingly, a recent report by Zhao and colleagues revealed that *ACE2* expression was concentrated in a small population (~1.4%) of AT2 cells [38]. Here, we further interrogated a relatively larger and more diverse repertoire of airway lineages, from both lung tumor (LUAD) tissues and multiple matched normal tissues per patient, and found that not only were expression levels of *ACE2* the highest in a minute fraction of AT2 cells (~2.2%, in close agreement with Zhao et al.’s report) but also that the greatest fraction of *ACE2*-positive cells was in cells of the malignant-enriched cluster. We also identified high expression of the serine proteases *TMPRSS2* and *TMPRSS4* in cells of alveolar and malignant-enriched clusters, respectively, the former of which also positively correlated with AT2 abundance. More recently, these proteases have been highlighted as additional host proteins mediating SARS-CoV-2 cleavage and internalization [7,10]. Interestingly, *TMPRSS2* and *TMPRSS4* had been previously ascribed diverse roles in mucosal pathobiology, including that of the lung, such as the activation of influenza and SARS-CoV viruses [39,40,41], promotion of lung fibrosis [42], as well as the induction of lung cancer stem-cell-like properties [43], growth, metastasis, and resistance to chemotherapy [44]. It is worthwhile to mention that patients with cancer, including lung malignancy, were shown to represent a population that is extremely vulnerable to COVID-19 [3,45]. It is plausible to surmise that a better understanding of the expression of SARS-CoV-2 receptors is highly warranted in lung cancer patients, particularly in LUADs, whose pathogenesis is linked to *ACE2*-expressing airway lineages (e.g., AT2), and also since LUADs arise in the lung periphery, a major site for the development of pneumonia in COVID-19 patients. Our finding on the expression of *ACE2* and *TMPRSS4* in lung cells enriched with malignant (adenocarcinoma) features may have implications for the management of COVID-19 in lung cancer, and clinical studies are warranted to explore this conjecture. Our analysis also revealed significantly elevated *ACE2* levels, among *ACE2*-positive AT2 and cells of the malignant-enriched cluster, in patients who were on hypertension treatment with losartan compared to those without antihypertensive therapy. These findings are in line with previous reports, suggesting that antihypertensive agents may augment *ACE2* expression [23], and further, may partially explain the increased prevalence of hypertension in COVID-19 patients [46]. We thus cannot construe the implications of this finding on COVID-19 management [46], and further studies (e.g., larger cohort) are warranted to better explore the association between treatment with antihypertensive drugs and expression of SARS-CoV-2 receptors in the lungs of LUAD patients.

While our study provides novel insights into the expression of SARS-CoV-2 entry factors in LUAD tissues, its results need to be interpreted with caution. Our patient cohort is limited in size, and our study was designed with a focus on the single-cell analysis of epithelial cells from LUAD specimens. Future studies interrogating larger and more diverse cohorts will provide more power to analyze the expression of SARS-CoV-2 factors by LUAD stage or across histological subtypes, as well as in individual patients and at the single-cell level. Furthermore, it is conceivable that expression patterns of *ACE2* and SARS-CoV-2 host proteases in malignancies of the upper airways may be different from the patterns observed in LUAD (i.e., a cancer of the peripheral lung), and this is supported by recent studies showing the expression of *ACE2* in secretory cells (such as those in the upper airway) [47,48], which we also noted in our limited cohort. Additional studies in more diverse lung cancer cohorts are thus needed to interrogate these suppositions.

We found that *ACE2*-positive compared to *ACE2*-negative AT2 cells exhibited increased levels of genes with crucial roles and expression features in lung pathological diseases. The hedgehog interacting protein *HHIP* was not only shown to play important roles in airway branching during lung development [24], but also single nucleotide polymorphisms of this gene are associated with increased risk for COPD [25], a pulmonary ailment characterized by chronic inflammation [49]. *FGG* coding for fibrinogen-gamma was shown to be induced by proinflammatory cytokines [27] and to be elevated in lung pneumonia and infection [26]. *C4BPA*, coding for C4BP and part of the complement system, was found to recognize and bind pneumonia-causing streptococci in the lung epithelium [28,29]. It is noteworthy that many patients with COVID-19 (e.g., those with severe disease) commonly display the same pathological phenotypes, namely lung inflammation, fibrosis, and pneumonia, linked to those *ACE2-*co-expressed genes. It is intriguing to suggest that perhaps this small population of *ACE2*-expressing cells may underlie the pathogenesis of severe ARDS, pneumonia, and respiratory failure in COVID-19 patients and, perhaps, particularly in those with LUAD. Our findings on SARS-CoV-2 receptor expression patterns in uninvolved normal lungs of patients with LUAD support the need for future studies comparing the expression of those genes between COVID-19 patients with and without LUAD. 

A notable finding in our study was the co-expression of pathogens, including viral- scavengers and receptors such as *CD36* and *DMBT1* in *ACE2*-positive AT2 cells. We also found that among all lung subsets, AT2 cells distinctly and markedly displayed the highest expression (fraction and level) of *DMBT1*. Further, *DMBT1* correlated with AT2 fractions in independent cohorts of bulk-sequenced lung tissues and positively correlated with *ACE2* in the AT2 compartment. DMBT1, also known as gp340, was shown to inhibit influenza A by binding to hemagglutinin on the virus [50]. DMBT1 was also shown to interact with surfactant protein D in alveolar cells to agglutinate and inhibit influenza A virus [31]. It has been suggested that the antiviral drug oseltamivir cooperates with innate immune proteins such as DMBT1 in the inhibition of lung epithelial cell infection by influenza A virus [50]. Interestingly soluble DMBT1 in saliva was shown to exert host defense roles (neutralization or inhibition of oral transmission) against influenza A virus [50] as well as HIV-1 [51]. DMBT1 was shown to specifically inhibit HIV-1 infectivity by binding to the virus envelope protein gp120, the same protein that binds to the CD4 receptor on host T cells [52]. In a manner similar to the ACE2-mediated entry route facilitating SARS-CoV-2 infection in the lung [7], DMBT1 was shown to aid in HIV-1 transcytosis across genital tract tissue [53]. The study by Stoddard and colleagues also demonstrated that HIV-1 transport can be inhibited by antibodies or peptides that block the interaction of DMBT1 with the HIV-1 envelope protein gp120 [53]. Given our findings on *DMBT1* co-expression with *ACE2* in lung AT2 cells, as well as its reported binding to multiple viruses, we suggest that targeting DMBT1 using soluble peptides [54] or by antibody-based neutralization may represent a viable strategy to counteract SARS-CoV-2 infection and ameliorate COVID-19.

## 5. Conclusions

Single-cell transcriptomic analysis of our lung epithelial single-cell cohort demonstrated that among all lung cell subsets, cells of AT2 and malignant-enriched clusters displayed the highest relative expression of the SARS-CoV-2 receptor *ACE2*, albeit at low cell fractions. Our data also highlight alveolar and malignant subsets that express the serine proteases *TMPRSS2* and *TMPRSS4* implicated in lung pathobiology, including cancer, which also act as SARS-CoV-2 coreceptors. We further found that the viral scavenger *DMBT1* is highly expressed in AT2 cells and correlates with *ACE2* in this compartment. Our study underscores epithelial cell populations in the lungs of LUAD patients that express SARS-CoV-2 receptors as well as genes involved in inflammatory lung pathological conditions and host defense, and, thus, points to potential targets underlying the development of COVID-19 in lung cancer patients.

## Data Availability

All sequencing data generated in this study are deposited in the European Genome-phenome Archive (EGAS00001005021).

## Supplementary Materials

The following are available online at https://www.mdpi.com/2072-6694/13/6/1250/s1, Figure S1: Single-cell expression of ACE2, TMPRSS2, and TMPRSS4 in cycling lung cells, Figure S2: Single-cell clustering of lung epithelial cells and analysis of ACE2, TMPRSS2, and TMPRSS4 expression in normal versus tumor LUAD TCGA samples, Figure S3: Single-cell expression of ACE2, TMPRSS2, and TMPRSS4 in AT2 subclusters, Figure S4: Single-cell expression of ACE2 in AT2 and malignant-enriched subsets of losartan-treated versus untreated patients, Figure S5: Single-cell expression of DMBT1 and the coronavirus receptors BSG and DPP4 in lung epithelial cells, Figure S6: Correlations between estimated AT2 cell fractions and *ACE2*, *DMBT1, TMPRSS2,* and *TMPRSS4* in TCGA normal lung samples of stage I or stages II and III LUADs, Table S1: Clinicopathological variables of the LUAD patients analyzed by scRNA-seq, Table S2: scRNA-seq quality metrics and number of EPCAM+ cells analyzed in tumor and multiple normal lung tissues, Table S3: Cell numbers of total and ACE2+ cells in epithelial subclusters.
