# Supplementary material for: Single-Cell Expression Landscape of SARS-CoV-2 Receptor ACE2 and Host Proteases in Normal and Malignant Lung Tissues from Pulmonary Adenocarcinoma Patients
Source: Cancers (Basel). 2021 Mar 12;13(6):1250. doi: 10.3390/cancers13061250 (PMC7998226; doi:10.3390/cancers13061250)
Supplement: Supplementary file 1 [file cancers-13-01250-s001.pdf]

Single-cell expression landscape of SARS-CoV-2 receptor *ACE2* and host proteases in human lung adenocarcinoma

ONLINE DATA SUPPLEMENT

SUPPLEMENTARY METHODS

Single-cell derivation and sorting

Following surgery, sample collection was performed by an experienced pathologist assistant (PB) starting with 2-3 normal lung tissues first and followed by the tumor sample, to minimize cross-contamination during collection. Normal samples were collected from up to 100 mm away from the tumor edge, and all sample sizes were in the range of 0.1 – 1 mm^3. Samples were immediately placed in ice-cold RPMI medium supplemented with 2% fetal bovine serum (FBS) and transported on ice for further processing. Tissues were washed in ice-cold Hank's balanced salt solution (HBSS) and extra-pulmonary airways and connective tissue was removed with scissors. Samples were transferred to dish on ice for mincing into 1mm^3 pieces and subsequent enzymatic digestion at 37°C with gentle rotation. Samples were digested in 3 ml of a cocktail containing Collagenase A (Sigma Aldrich), Collagenase IV (Thermo Fisher Scientific), DNase I, (Sigma Aldrich), Dispase II (Sigma Aldrich), Elastase 1 (Thermo Fisher Scientific) and Pronase (Sigma Aldrich), as previously described [1]. 10 minutes into the digestion reaction, the digest was mixed for further disaggregation, followed by an additional 10 min incubation at 37°C. Samples were then filtered, washed with ice-cold HBSS, and centrifuged. Red blood cells (RBCs) were removed from the cell pellet by incubation in ice-cold ACK lysis buffer (A1049201, Thermo Fisher Scientific). RBC lysis reaction was stopped by adding ice-cold FBS, and samples were centrifuged and re-suspended in ice-cold phosphate-buffered saline (PBS) with 2% FBS,

filtered, and cells from patients 2 - 5 (P2-P5) were additionally stained with anti-EPCAM-PE (347198, BD Biosciences; 1:50 dilution) for 30 minutes with gentle rotation at 4°C. Cells from all patients were washed, stained with SYTOX Blue viability dye (S34857, Life Technologies) in PBS 2% FBS, and processed on a fluorescence activate cell sorting (FACS) Aria I instrument. Viable singlets (SYTOX Blue negative, P1), or EPCAM+ and EPCAM- viable singlets (P2-P5) were sorted into PBS 2% FBS, and immediately washed, filtered and enumerated by Trypan Blue before loading individual fractions on 10X Chromium Next GEM Chips G (1000127, 10X Genomics) according to manufacturer's instructions.

Preparation of single-cell gene expression libraries

Gel beads-in-emulsion (GEMs) generated using Chromium Next GEM Single Cell 5' Gel Bead Kit v1.1 (1000169, 10X Genomics) were recovered from individual chip channels and single-cell 5' gene expression libraries were constructed using the Chromium Next GEM Single Cell 5' Library kit (1000166, 10X Genomics) according to the manufacturer's protocol. 10X-barcoded full-length cDNA was recovered from the barcoded GEMs following dissociation and magnetic bead clean-up (Dynabeads MyOne Silane, Thermo Fisher Scientific). Following PCR amplification of cDNA and quality control (QC) using Bioanalyzer High Sensitivity DNA kit (Agilent), up to 50 ng of amplified cDNA was used to construct gene expression libraries. cDNA was enzymatically fragmented, size-selected, and subject to end-repair, A-tailing, adaptor ligation, and sample index PCR using Single Index Kit T Set A (10X Genomics). Illumina-ready barcoded gene expression libraries were assessed using Bioanalyzer High Sensitivity DNA kit (Agilent) and Qubit dsDNA High Sensitivity Assay Kit (Thermo Fisher Scientific), pooled, denatured and diluted as recommended for sequencing on the Illumina NovaSeq 6000 platform.

scRNA-seq data quality control

Detailed QC metrics including UMI count per barcode, number of genes detected and mitochondrial gene rate, were generated and evaluated. Genes detected in < 3 cells and cells where < 200 genes had non-zero counts were filtered out and excluded from subsequent analysis. Low quality cells where >15% of the read counts originated from the mitochondrial genome were also discarded. In addition, cells with >6,000 detected genes were discarded to remove likely doublet or multiplet captures. Possible batch effects were evaluated and corrected using Harmony [2].

TCGA data analysis

Expression analysis of select genes was done in bulk mRNA-seq expression dataset (normalized) of primary LUADs with matched normal lung tissues (n = 52) generated by The Cancer Genome Atlas (TCGA) Program on LUAD [3] and downloaded from the NCI Cancer Genomic Data Commons (NCI-GDC: https://gdc.cancer.gov). The mRNA-seq expression data were processed and normalized by the NCI-GDC bioinformatics team using their transcriptome analysis pipeline. Deconvolution of AT2 cell abundance in TCGA LUAD cohort was performed by incorporating AT2 signature genes defined by our study into MCP-counter, an R package that permits the quantification of the absolute abundance of cell populations in heterogeneous tissues from transcriptome data [4].

SUPPLEMENTARY TABLES

Table S1: Clinicopathological variables of the LUAD patients analyzed by scRNA-seq

Patient (P)	LUAD stage	Histological pattern	Age	Sex	Ethnicity	Losartan treatment
P1	IIIA	acinar adenocarcinoma	70	Female	White or Caucasian	Yes
P2	IB	invasive mucinous adnocarcinoma	73	Female	White or Caucasian	Yes
P3	IIIA	invasive mucinous adnocarcinoma	58	Female	White or Caucasian	No
P4	IIB	acinar adenocarcinoma	64	Female	White or Caucasian	No
P5	IB	papillary adenocarcinoma	71	Male	White or Caucasian	Yes

LUAD: lung adenocarcinoma

Table S2: Cell numbers of total and ACE2+ cells in epithelial subclusters

Cluster	Cell Number	ACE2+ Cells
Alveolar progenitor	5516	41
AT1	10775	31
AT2	27235	607
Basal	5119	49
Bronchioalveolar	2336	6
Ciliated	3247	18
Club and secretory	4624	110
Ionocyte	64	0
Proliferating basal	447	10
Malignant-enriched	10704	370

AT1: alveolar type 1; AT2: alveolar type 2

SUPPLEMENTARY REFERENCES

1. Slyper, M., et al., *A single-cell and single-nucleus RNA-Seq toolbox for fresh and frozen human tumors.* Nature Medicine, 2020. **26**(5): p. 792-802.
2. Korsunsky, I., et al., *Fast, sensitive and accurate integration of single-cell data with Harmony.* Nat Methods, 2019. **16**(12): p. 1289-1296.
3. Cancer Genome Atlas Research, N., *Comprehensive molecular profiling of lung adenocarcinoma.* Nature, 2014. **511**(7511): p. 543-50.
4. Becht, E., et al., *Dimensionality reduction for visualizing single-cell data using UMAP.* Nat Biotechnol, 2018.

SUPPLEMENTARY FIGURE LEGENDS

Figure S1. Single-cell expression of *ACE2*, *TMPRSS2*, and *TMPRSS4* in cycling lung cells. Uniform manifold approximation and projection (UMAP) embedding showing cycling lung cells from all 19 samples (35 single-cell expression libraries). Cells are color-coded by expression level of *ACE2*, *TMPRSS2*, or *TMPRSS4*.

Figure S2. Single-cell clustering of lung epithelial cells and analysis of *ACE2, TMPRSS2, and TMPRSS4* expression in normal versus tumor LUAD TCGA samples. A, Bubble plots showing cell fractions with scaled expression of airway lineage markers for the identified lung epithelial cell clusters. **B,** UMAP plot of all epithelial cells labeled by sample type. **C,** Analysis of *ACE2*, *TMPRSS2*, and *TMPRSS4* expression in matched tumor and normal samples from TCGA LUAD cohort. LUAD: lung adenocarcinoma; AT1: alveolar type 1; AT2: alveolar type 2

Figure S3. Single-cell expression of *ACE2*, *TMPRSS2*, and *TMPRSS4* in AT2 subclusters. A, UMAP showing unsupervised clustering of AT2 cells. Cells are colored by assigned AT2 subcluster. **B-D,** Bar plots showing fraction of AT2 cells (percentage of each cluster) expressing *ACE2* **(B)**, *TMPRSS2* **(C)** and *TMPRSS4* **(D)** among AT2 clusters with highest fractions of cells positive for each gene. Absolute number of cells positive for each gene and within each analyzed AT2 cluster are indicated on top of the corresponding bars. Color indicates average expression in AT2 cells positive for the gene of interest.

Figure S4. Single-cell expression of *ACE2* in AT2 and malignant-enriched subsets of losartan-treated versus untreated patients. A, Bar plots showing *ACE2* expression frequency and levels in AT2 or malignant-enriched subsets and in losartan-treated versus untreated patients. Color indicates average expression in cells positive for *ACE2*. **B,** Violin plots showing

significant up-regulation of *ACE2* expression in *ACE2*+ AT2 or malignant-enriched cells of losartan-treated patients compared to untreated patients. FC: Fold change.

Figure S5. Single-cell expression of *DMBT1* and the coronavirus receptors *BSG* and *DPP4* in lung epithelial cells. A-B Bar plots showing expression frequency and levels of *DMBT1, BSG*, and *DPP4* in the 10 identified lung epithelial cell clusters **(A)** and among the 5 AT2 clusters **(B).**

Figure S6. Correlations between estimated AT2 cell fractions and *ACE2*, *DMBT1, TMPRSS2,* and *TMPRSS4* in TCGA normal lung samples of stage I or stages II and III LUADs. Correlations were statistically analyzed using Pearson's correlation coefficient.

Figure S1

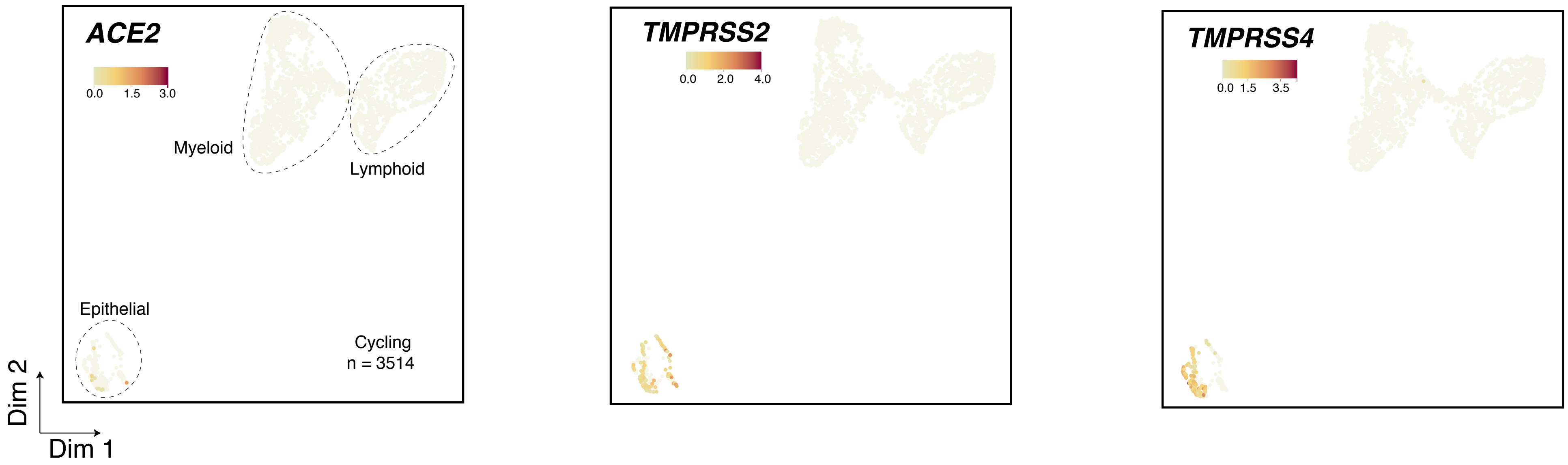

ACE2
0.0 1.5 3.0
Myeloid
Lymphoid
Epithelial
Cycling
n = 3514
Dim 2
Dim 1
TMPRSS2
0.0 2.0 4.0
TMPRSS4
0.0 1.5 3.5

Figure S2

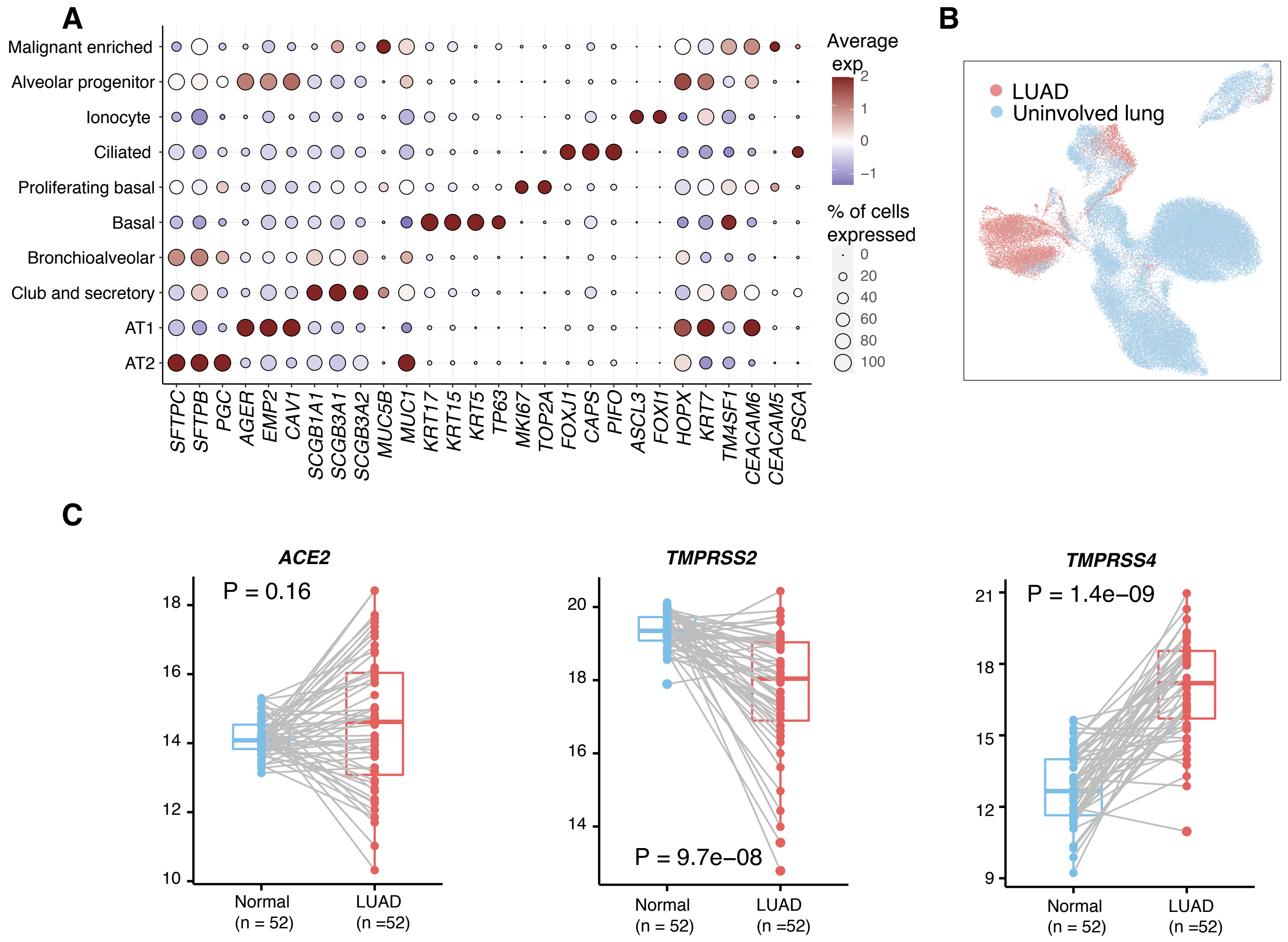
A
Malignant enriched
Alveolar progenitor
Ionocyte
Ciliated
Proliferating basal
Basal
Bronchioalveolar
Club and secretory
AT1
AT2
SFTPC SFTPB PGC AGER EMP2 CAV1 SCGB1A1 SCGB3A1 SCGB3A2 MUC5B MUC1 KRT17 KRT15 KRT5 TP63 MKI67 TOP2A FOXJ1 CAPS PIFO ASCL3 FOXI1 HOPX KRT7 TM4SF1 CEACAM6 CEACAM5 PSCA
Average exp
2
1
0
−1
% of cells expressed
0
20
40
60
80
100
B
LUAD
Uninvolved lung
C
ACE2
P = 0.16
TMPRSS2
P = 9.7e−08
TMPRSS4
P = 1.4e−09
Normal (n = 52)
LUAD (n =52)

Figure S3

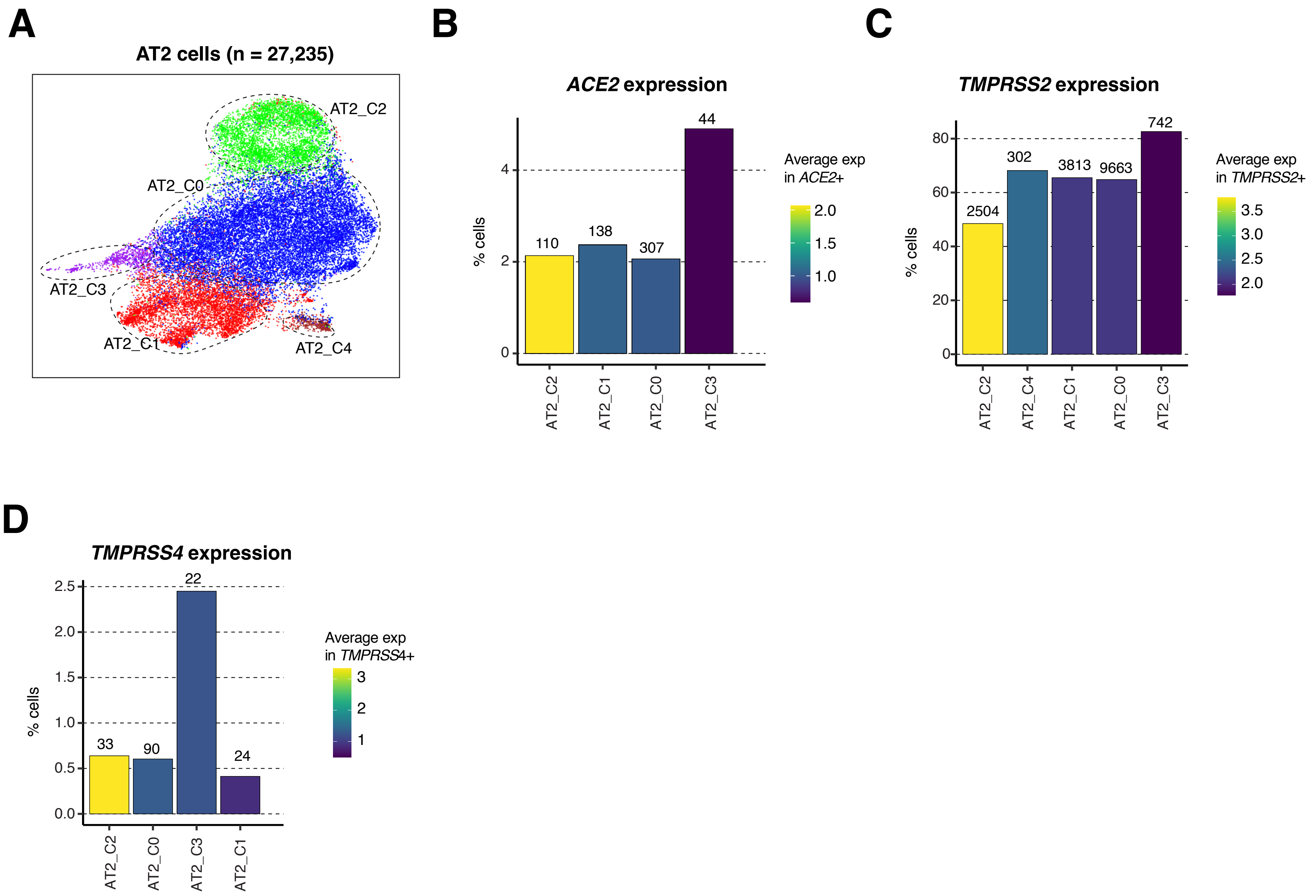
A
AT2 cells (n = 27,235)
AT2_C2
AT2_C0
AT2_C3
AT2_C1
AT2_C4
B
ACE2 expression
% cells
0
2
4
110
138
307
44
AT2_C2
AT2_C1
AT2_C0
AT2_C3
Average exp
in ACE2+
2.0
1.5
1.0
C
TMPRSS2 expression
% cells
0
20
40
60
80
2504
302
3813
9663
742
AT2_C2
AT2_C4
AT2_C1
AT2_C0
AT2_C3
Average exp
in TMPRSS2+
3.5
3.0
2.5
2.0
D
TMPRSS4 expression
% cells
0.0
0.5
1.0
1.5
2.0
2.5
33
90
22
24
AT2_C2
AT2_C0
AT2_C3
AT2_C1
Average exp
in TMPRSS4+
3
2
1

Figure S4

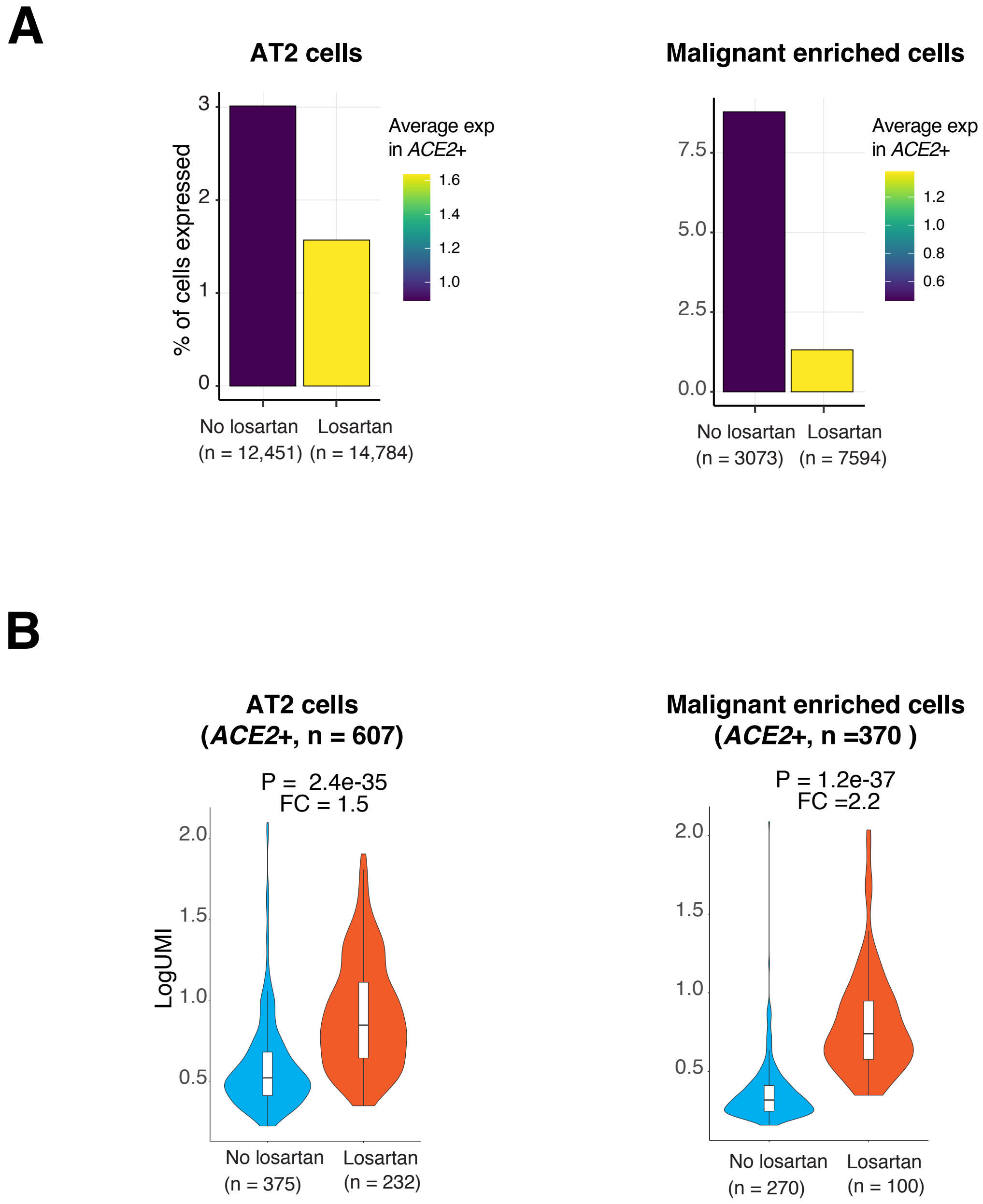
A
AT2 cells
% of cells expressed
3
2
1
0
Average exp
in ACE2+
1.6
1.4
1.2
1.0
No losartan
(n = 12,451)
Losartan
(n = 14,784)
Malignant enriched cells
7.5
5.0
2.5
0.0
Average exp
in ACE2+
1.2
1.0
0.8
0.6
No losartan
(n = 3073)
Losartan
(n = 7594)
B
AT2 cells
(ACE2+, n = 607)
P = 2.4e-35
FC = 1.5
LogUMI
2.0
1.5
1.0
0.5
No losartan
(n = 375)
Losartan
(n = 232)
Malignant enriched cells
(ACE2+, n =370)
P = 1.2e-37
FC =2.2
2.0
1.5
1.0
0.5
No losartan
(n = 270)
Losartan
(n = 100)

Figure S5

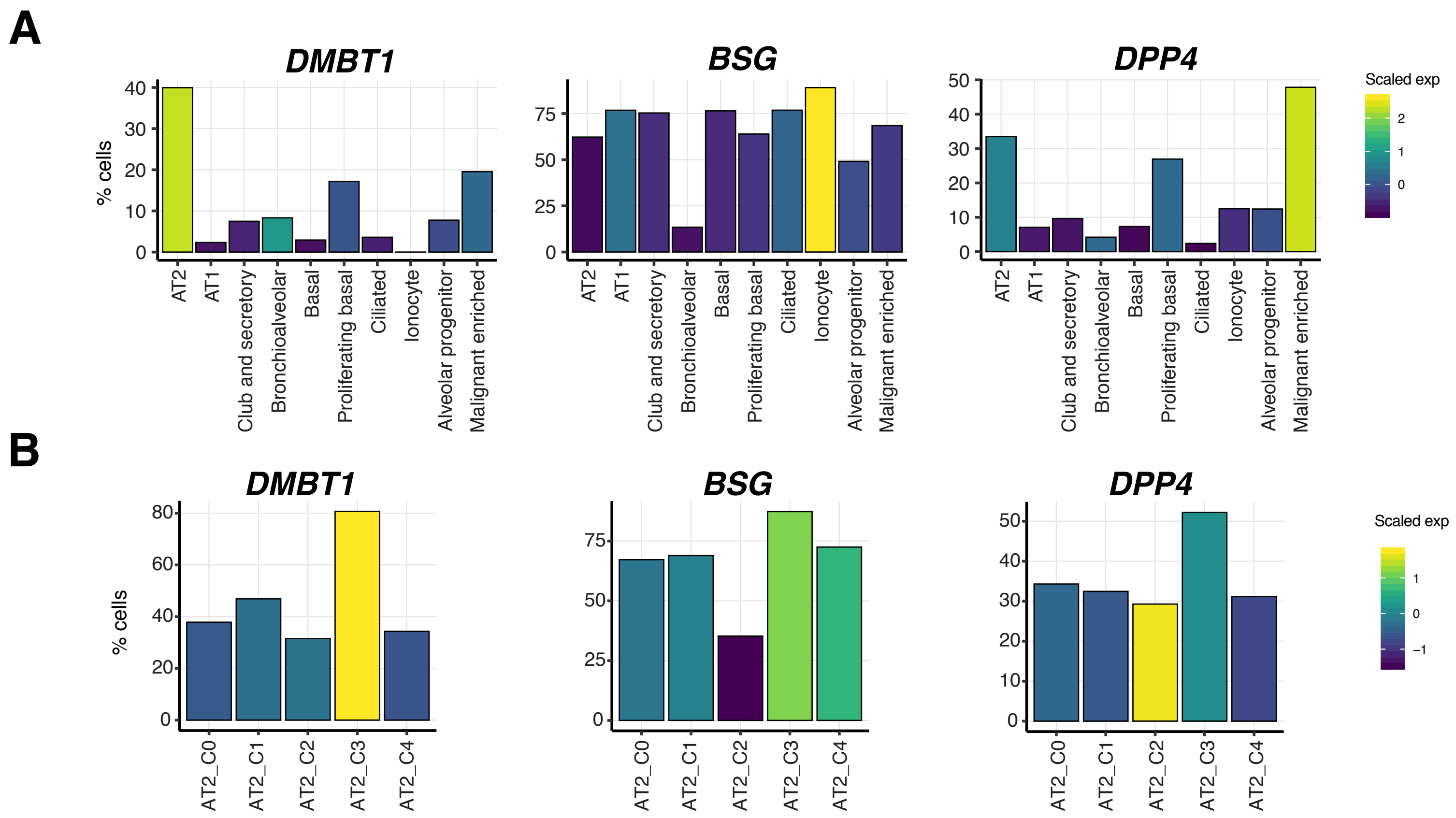
A
DMBT1
BSG
DPP4
% cells
AT2
AT1
Club and secretory
Bronchioalveolar
Basal
Proliferating basal
Ciliated
Ionocyte
Alveolar progenitor
Malignant enriched
Scaled exp
B
AT2_C0
AT2_C1
AT2_C2
AT2_C3
AT2_C4

Figure S6

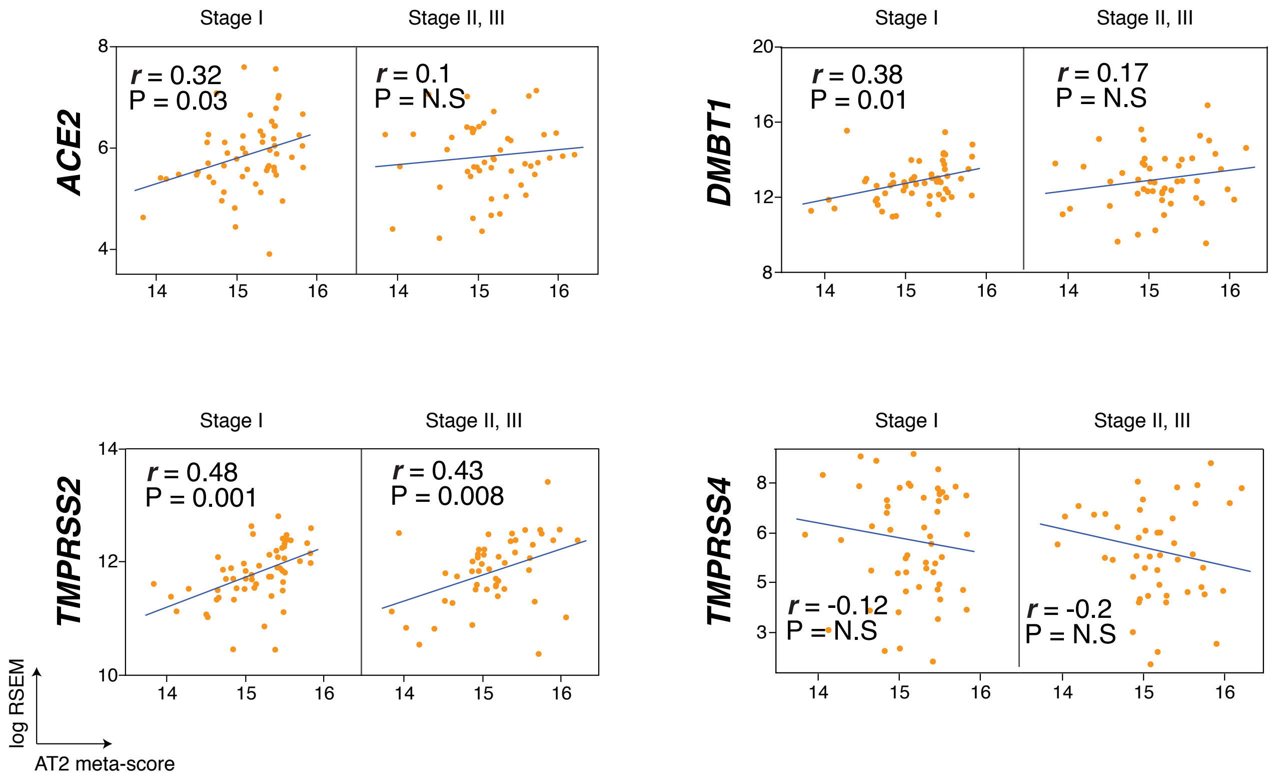
Stage I
Stage II, III
ACE2
r = 0.32
P = 0.03
r = 0.1
P = N.S
DMBT1
r = 0.38
P = 0.01
r = 0.17
P = N.S
TMPRSS2
r = 0.48
P = 0.001
r = 0.43
P = 0.008
TMPRSS4
r = -0.12
P = N.S
r = -0.2
P = N.S
log RSEM
AT2 meta-score
